# Curcumin/Usnic Acid-Loaded Electrospun Nanofibers Based on Hyaluronic Acid

**DOI:** 10.3390/ma13163476

**Published:** 2020-08-07

**Authors:** Petr Snetkov, Svetlana Morozkina, Roman Olekhnovich, Thi Hong Nhung Vu, Maria Tyanutova, Mayya Uspenskaya

**Affiliations:** Institute BioEngineering, ITMO University, Kronverkskiy prospekt, 49A, 197101 St. Petersburg, Russia; Morozkina.Svetlana@gmail.com (S.M.); r.o.olekhnovich@itmo.ru (R.O.); vuhongnhungs@itmo.ru (T.H.N.V.); tyanutovaM@itmo.ru (M.T.); mv_uspenskaya@itmo.ru (M.U.)

**Keywords:** biomaterials, curcumin, electrospinning, fiber technology, hyaluronic acid, usnic acid

## Abstract

Hyaluronic acid, curcumin, and usnic acid are separately utilized as effective biological agents in medicine, and materials based on its blend are considered to have wider therapeutic effects than individual ones. In this study, for the first time, native hyaluronic acid-based fibers containing curcumin and usnic acid with an average diameter of 298 nm were successfully prepared by the electrospinning technique and characterized. Additionally, unstable and hydrophobic curcumin and usnic acid were loaded into the hydrophilic hyaluronic acid matrix without utilizing the activating (catalyzing) agents, resulting in the formation of an electrospinnable solution. Only the binary mixture deionized water—dimethyl sulfoxide (50:50)—was used as solvent. The presence of small amounts of dimethyl sulfoxide in the fibrous materials was expected to provide the materials with local anesthetic and antiseptic activity. The effect of electric voltage on the electrospinning process, diameter, and morphology of hyaluronic acid/curcumin/usnic acid fibers was investigated in detail. The impact of curcumin and usnic acid on the stability of fiber formation was observed. The investigation of fibers based on pure hyaluronic acid without additional polymers and with active pharmaceutical ingredients will lay the groundwork for the development of highly effective wound dressings and new drug delivery scaffolds.

## 1. Introduction

Hyaluronic acid as an unbranched polymer composed of repeating disaccharide units of 1-4-D-glucuronic acid and 1-3-N-acetyl-D-glucosamine, being a major component of the intracellular, extracellular, and pericytial matrixes, is one of the attractive native polymers. Due to the unique biocompatible, biodegradable, non-immunogenic, and non-allergic properties it could be applied for the fabrication of a variety of promising and advanced biomedical applications: films [1], nanoparticles [2], nanofibers [3], eye drops [4], non-adhesive bandage [5], dermal fillers [6], etc. Preparation of nanofiber is known as an interesting and challenging scientific subject, as fibrous materials based on hyaluronic acid will have great advantages compared with the polymer films due to the high sponge, specific surface area of fibrous structures, and higher permeability.

Nanofibers could be formed from polymer solutions by the electrospinning technique, which enforces following certain rules. Thus, the obtaining of fibrous materials based on hyaluronic acid from aqueous solutions by electrospinning is a complex process due to the insolubility of hyaluronic acid in organic solvents, which is necessary for the electrospinning technique. Aqueous solutions of hyaluronic acid have high levels of electrical conductivity and viscosity, coupled with low volatility. All the above-mentioned factors hinder the electrospinning process [7,8,9,10]. For example, wet nanofibers with the unevaporated water could function as conductors among the electrodes, leading to the probability of an “electric breakdown” [3].

To solve this problem, many researchers obtain polymer fibers using modifying (carrier) polymers, such as polyethylene oxide (PEO) [11,12,13,14,15], polyvinyl alcohol (PVA) [16,17], polyamide [18], collagen [10,19], chitosan [20,21], silk fibroin [22], and gelatin as surfactant [23]. Interestingly, Zhao Y. et al. [17] initially obtained polyvinyl alcohol/polyethyleneimine nanofibers crosslinked by glutaric aldehyde following modification by hyaluronic acid. However, the concentration of hyaluronic acid in the resulting materials is smaller than the carrier polymer concentration, therefore materials based on them do not have regenerative and anti-inflammatory properties. Thus, the application of fibrous materials based on hyaluronic acid containing PEO or PVA is not expedient for medicine.

Another method of obtaining nanofibers from hyaluronic acid without carrier polymers is to use binary and ternary aqueous−organic solutions [23,24,25,26]. Often, such solutions contain toxic solvents, such as dimethylformamide, which residuals in fibrous materials having a negative irritating effect on tissues. By contrast, residual amount of dimethyl sulfoxide as pharmaceutical agent is expected to add additional anti-inflammatory and local anesthetic properties to nanofibers [27]. The hydrochloric acid is also utilized [7,8]. Interestingly, organic or mineral acids are utilized to improve the hyaluronic acid solutions’ conductivity, which allows for obtaining nanofibers [11,20,26]. However, even a small amount of acid leads to depolymerization of the hyaluronic acid [28].

**Figure 1:**
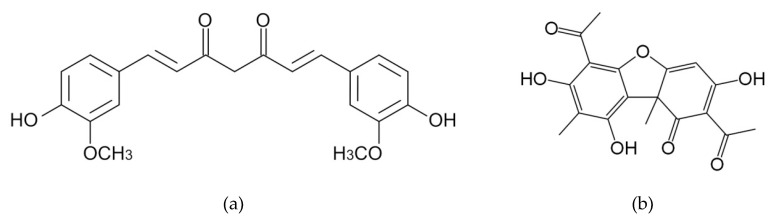
Chemical structure of curcumin (**a**) and usnic acid (**b**).

The loading of therapeutic pharmaceutical agents into the fibers represents a more interesting task than obtaining fibers based on native hyaluronic acid. For example, the fibrous materials based on hyaluronic acid and water-soluble kanamycin [12] and ibuprofen [15] were successfully obtained. Note that naturally occurring biologically active substances are more attractive because of their widespread use, high efficacy, and low adverse effects. However, such biomedical substances have a hydrophobic nature which hinders the usability. Particular attention is paid to curcumin from *Curcuma longa* (Figure 1a) due to its anti-inflammatory, antimicrobial, antioxidant, antiviral and anticarcinogenic activity [29,30]. 

Usnic acid (Figure 1b) from the species of *Usnea*, *Cladonia*, *Parmelia*, *Ramalina*, *Lecanora*, *Evernia*, *Thamnolias*, and other lichens also comes into notice due to unique properties similar to curcumin [31,32,33]. Interestingly, usnic acid possesses antimalarial [34] and antituberculosis activity [33].

Unfortunately, the known polymer materials based on hyaluronic acid and curcumin were obtained by using the irritant, hazard, and toxic activating (catalyzing) agents such as 1,3-dicyclohexylcarbodiimide (DCC) and 4-dimethylaminopyridine (DMAP) [35,36,37]. By contrast, usnic acid is successfully utilized for obtaining hyaluronic acid compositions and nanoparticles [38,39] without such agents. Note that there are not enough studies addressing usnic acid/hyaluronic acid compositions. Moreover, there are no reports of obtaining nanofibers based on hyaluronic acid with a blend of curcumin and usnic acid.

In this paper, we describe the first example of curcumin/usnic-acid-loaded nanofibers based on native hyaluronic acid obtained without the carrier polymers and modifiers in a mixture of water-DMSO solvents. DCC and DMAP were not utilized at all. The electrospinning technique, absence of toxic regents, and natural biomedical additives are expected to obtain non-toxic and biodegradable materials with high opportunity for wound healing, tissue engineering, and drug delivery [40,41,42].

## 2. Materials and Methods

### 2.1. Materials

Sodium hyaluronate HA-T (MW about 1.30 MDa, glucuronic acid content 45%, protein content 0.05%) was obtained from Bloomage Freda Biopharm CO., LTD (Jinan, China). Dimethyl sulfoxide (DMSO, 99.5% ACS, MW = 78.13 g/mol) was purchased from JSC EKOS-1 (Russian Federation). Curcumin (MW = 368.38 g/mol) from *Curcuma longa* (Turmeric) and usnic acid (MW = 344.32 g/mol) from *Usnea dasypoga* were supplied by Sigma-Aldrich (St. Louis, MO, USA). All materials were used as received without additional purification. Deionized water was obtained from the laboratory distillation unit.

Sodium hyaluronate was used as biopolymer matrix for electrospun nanofibers. DMSO was utilized as a co-solvent to decrease the electrical conductivity of the polymer solution and to improve the electrospinning process. Curcumin and usnic acid were used as biologically active substances having a natural origin.

### 2.2. Electrospinning Polymer Solutions

Sodium hyaluronate HA-T was dissolved in a distilled water/DMSO binary solvent system with a volume ratio 1:1 to obtain 1.9 wt.% solution [27]. Polymer solutions were mixed at 50 °C for 24 h using the magnetic stirrer. Mixed solutions were put on hold for at least 60 min at room temperature for splatter dashing and balancing. The molecular ratio of sodium hyaluronate monomeric unit to curcumin was varied from 2 to 25. The molecular ratio of curcumin to usnic acid was varied from 1 to 2.

### 2.3. Electrospinning Technique

**Figure 2:**
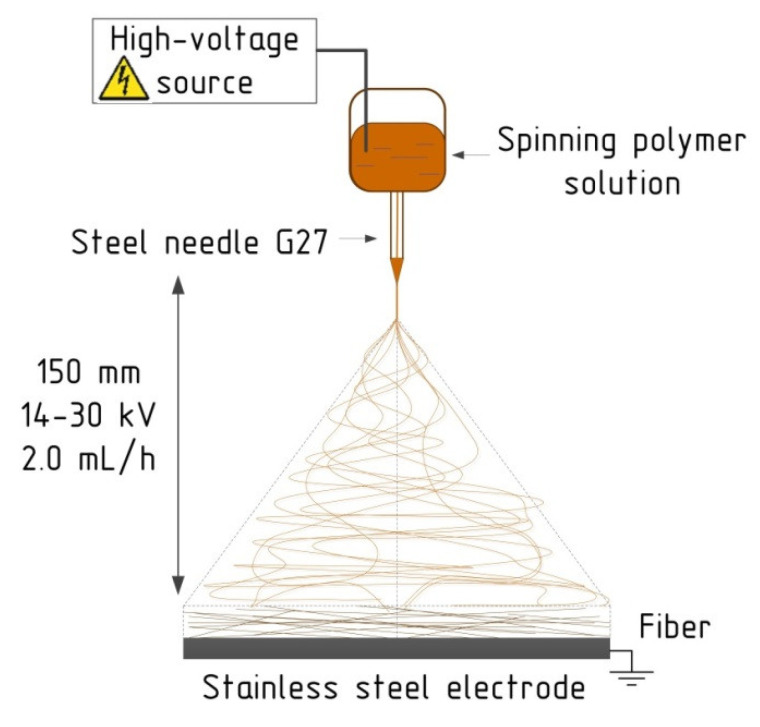
The schematic representation of electrospinning process and operating parameters.

Fiber formation was performed by utilizing the electrospinning system NANON-01A (MECC CO., LTD., Fukuoka, Japan). The principal scheme of the electrospinning process is demonstrated in Figure 2 and was considered in detail earlier [3].

Electrospinning was performed at a temperature of 21.0 ± 1.5 °C and a relative humidity of 30 ± 3%. Technological parameters were as follows: electric voltage from 14 to 30 kV; feed rate 2.0 mL/h; traverse speed 10 mm/s, 27G steel needles; plate stainless steel collector 150 mm × 200 mm (L × B); distance between needle and electrode of 150 mm. Electrospinnability of the obtained solutions was identified by the occurrence and shapes of the Taylor Cone and the jet path (length of the straight segment of a jet and the envelope cone), as was the stability of the process. The electrospinning process was undertaken at the specimen glasses 26 mm × 76 mm × 1 mm (L × B × H) for 5 min, followed by drying in the chamber for 10 min.

### 2.4. Morphology and Diameters of Nanofibers

For preliminary characterization, the morphology and diameters of electrospun fibers based on hyaluronic acid/curcumin/usnic acid the measuring optical microscope Olympus STM6 (OLYMPUS Corporation, Tokyo, Japan) was used. Differentially interferential contrasting technique (DIC) was utilized to emphasize the colorfulness and contrast of obtained fibers.

For detailed analysis, the scanning electronic microscope (SEM) MERLIN (Carl Zeiss) was utilized. The electron high tension (EHT) voltage was equal to 0.5 kV, signal A = SE2, working distance from 2.3 to 2.4 mm. Note that the samples were not evaporated by carbon or aurum, and were scanned as received.

For analysis and measurement, the fiber diameter on obtained microphotographs program ImageJ (National Institutes of Health, Bethesda, MD, USA) was used [43].

### 2.5. Statistical Analysis

The diameter distribution of obtained nanofibers was estimated by OriginPro 2019b (OriginLab Corporation, Northampton, MA, USA). For measuring diameter distribution, several images were used.

## 3. Results and Discussion

### 3.1. Solutions Electrospinnability

In this research, curcumin/usnic-acid-loaded nanofibers based on hyaluronic acid were obtained by electrospinning with a hyaluronic acid concentration of 1.9 wt.% and with a water–DMSO volume ratio of 50:50. The process of electrospinning solutions of hyaluronic acid containing curcumin and usnic acid is more stable than the process of electrospinning solutions of hyaluronic acid alone. Interestingly, a visual configuration of the envelope cone in the presence of curcumin and usnic acid was wider than without it. Moreover, there are no straight segments of a jet: the whipping area starts upon the tip of the needle. Note that all solutions have high levels of spinnability and storage stability under ambient conditions. However, noticeable process differences between the solutions with various molecular ratios of components are not observed.

### 3.2. The Influence of the Voltage Level

**Figure 3:**
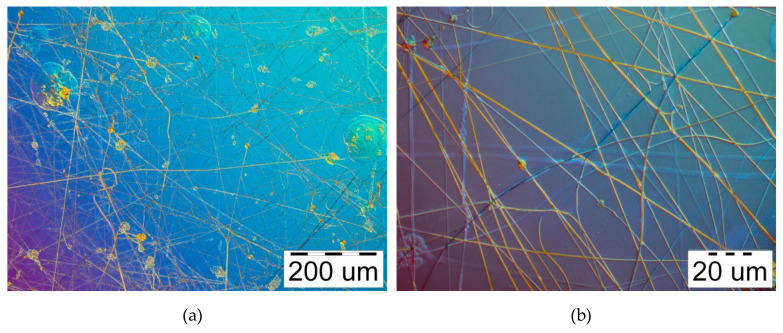
Microphotographs of curcumin/usnic acid-loaded hyaluronic acid fibers electrospun from the polymer solutions under 22 kV: (**a**) magnification 100×; (**b**) magnification 1000×.

**Figure 4:**
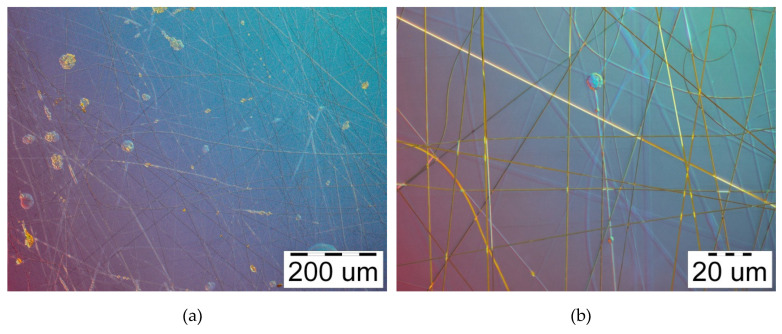
Microphotographs of curcumin/usnic acid-loaded hyaluronic acid fibers electrospun from the polymer solutions under 28 kV: (**a**) magnification 100×; (**b**) magnification 1000×.

The initial formation of the nanofibers begins already with a voltage of 14–16 kV, but under these conditions electrospinning was very unstable. Sudden flashes and individual large drops jetting off from the needle were observed. The process of obtaining of curcumin/usnic acid-loaded fibers based on hyaluronic acid is stable at 20–22 kV. However, many defects, such as small beads, branches, twisting, drops, etc., were seen. The above deviations are shown in Figure 3a,b. The number of some defects slightly decreases with an increase in electric voltage to 26–28 kV, as can be seen in the microphotographs presented in Figure 4a,b. A further increase in electrical voltage to 30 kV does not lead to a significant improvement in the electrospinning process.

### 3.3. SEM Analysis

**Figure 5:**
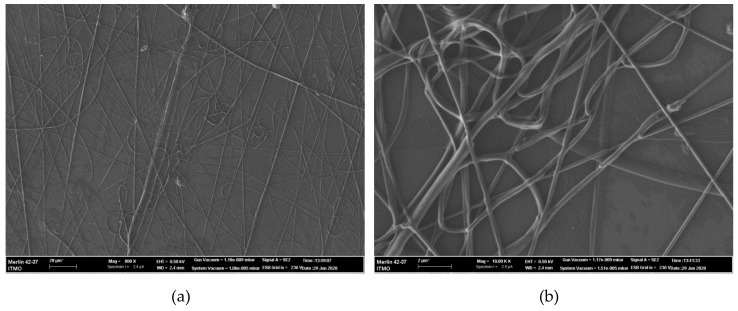
SEM images of curcumin/usnic acid-loaded hyaluronic acid fibers electrospun from the polymer solutions under 22 kV: (**a**) magnification 1000×; (**b**) magnification 10,000×.

**Figure 6:**
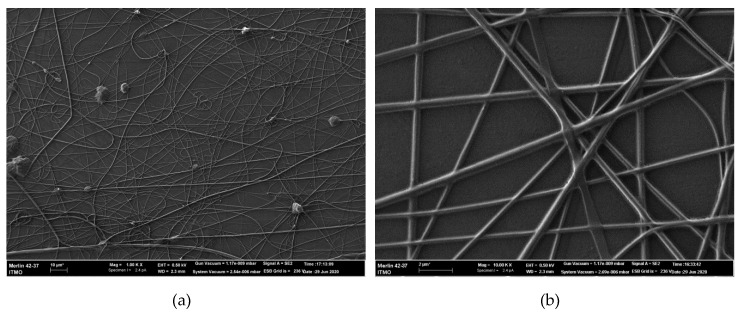
SEM images of curcumin/usnic acid-loaded hyaluronic acid fibers electrospun from the polymer solutions under 28 kV: (**a**) magnification 1000×; (**b**) magnification 10,000×.

SEM images for the nanofibers obtained under 22 and 28 kV are shown in Figure 5 and Figure 6, respectively. SEM photomicrographs were obtained at two magnifications: 1000× (a) and 10000× (b). The nanofibers morphology can be analyzed in detail with the SEM images. Firstly, due to less stability of the electrospinning process, the sample formed under 22 kV has less covering density than the sample obtained under 28 kV. Secondly, nanofibers obtained under 22 kV have more knots and tangles (see an example of such a defect in Figure 5b) than similar ones formed under increased voltage. By contrast, the nanofibers obtained under 28 kV have more individual drops, which could be related to the high level of feed rate. Interestingly, the formation of agglutinated fibers shown in Figure 6b is connected to the level of relative humidity: to obtain electrospun separated hyaluronic-acid-based nanofibers without agglutination, it is recommended to set the relative humidity below 8% [44], which is difficult to accomplish.

### 3.4. Summary Characterization

The summarized information of electrospinning process stability and obtained curcumin/usnic acid-loaded hyaluronic acid fibers morphology is demonstrated in Table 1. Note that the diameter distributions were obtained by ImageJ analysis of SEM photomicrographs.

**Figure 7:**
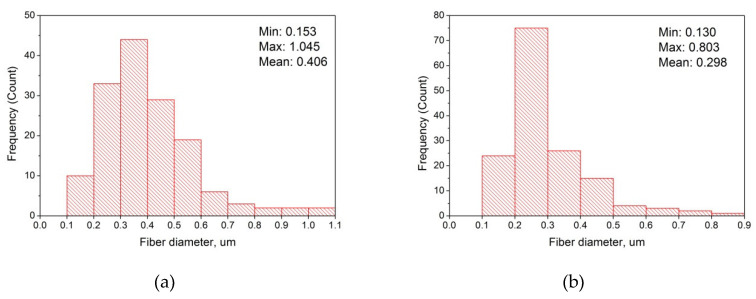
Diameter distribution of curcumin/usnic acid-loaded fibers based on hyaluronic acid: (**a**) under 22 kV; (**b**) under 28 kV. Note, than for measuring diameter distribution the several images were utilized.

Moreover, as shown in Figure 7a,b polymeric fibers fabricated under lower voltage have wider diameter distribution (from 0.153 to 1.045 μm) and higher mean diameter (0.406 μm) than fibers obtained under higher voltage (0.130–0.803 and 0.298 μm, respectively). This tendency towards nanofibers based on the hyaluronic acid corresponds to previous studies [45,46,47].

## 4. Conclusions

In this research, biopolymer fibers based on native hyaluronic acid with curcumin and usnic acid as active substances were, for the first time, successfully obtained without utilizing the carrier polymers by electrospinning from distilled water/DMSO solvent systems at room temperature. The mean nanofibers’ diameter is 298 nm. The loading of the hydrophobic curcumin and usnic acid into hydrophilic hyaluronic acid matrix was performed without utilizing toxic chemical agents such as DCC and DMAP. It is supposed that the absence of the above-mentioned catalyst reagents can provide the biocompatibility of materials based on curcumin/usnic acid-loaded hyaluronic acid. Moreover, the possible presence of DMSO in residual amounts in the fibrous materials is expected to enhance the anti-inflammatory properties and local analgesic and antiseptic activity of the fibers.

During the electrospinning process, the effect of the electric voltage as the main influencing parameter was demonstrated. It was found that the prepared solutions are easily electrospun in spite of the molecular ratio of hyaluronic acid and biologically active agents. This technology of curcumin/usnic-acid-loaded hyaluronic acid fibers obtainment significantly broadens the application of the electrospun fibers filled by pharmacological agents in modern biomedical systems, such as wound dressings, ambustial materials and drug delivery scaffolds.

## Figures and Tables

**Table 1:** Characterization of the electrospinning and curcumin/usnic acid-loaded fibers obtained.

Applied Voltage (kV)	Diameter of Fibers Obtained (μm)			Characterization	
	Min	Max	Mean	Fibers	Electrospinning
16	**-**	**-**	**-**	Drops	Unstable
22	0.153	1.045	0.406	Presence of a lot of defects: small beads, branches, curling, blobs, knots, tangles, etc.	Stable
28	0.130	0.803	0.298	Presence of individual small droplets, polymer clots and fiber curling	Very stable
